# EHMTI-0264. Differences of some cytochemical parameters in persons with and without migraine

**DOI:** 10.1186/1129-2377-15-S1-E26

**Published:** 2014-09-18

**Authors:** A Plinta, I Logina, P Tretjakovs, R Erts, G Bahs

**Affiliations:** 1 Doctoral studies Dept., RSU, Riga, Latvia; 2 Riga Stradins University Department of Neurology and Neurosurgery, RSU, Riga, Latvia; 3 Riga Stradins University Department of Human Physiology and Biochemistry, RSU, Riga, Latvia; 4 Riga Stradins University Department of Physics, RSU, Riga, Latvia; 5 Riga Stradins University Department of Family Medicine, RSU, Riga, Latvia

## Background

Diagnosis of migraine currently based only on clinical symptoms and exclusion of secondary headache. Useful screening method of migraine is still unmet medical need.

## Aim

Comparison of some cytochemical parameters in persons, suffering from migraine and healthy volunteers, and evaluate sensitivity of each parameter in case of significant difference between both groups.

## Materials and methods

49 persons suffering from migraine, only women (42,1 ± 9,3 y.o.) and 30 gender, age and body mass index matched healthy volunteers (38,2 ± 5,5 y.o.) were included in trial. Cytochemical parameters were evaluated: interferon gamma (IFN-g), interleukin-10 (IL-10), interleukin-8 (IL-8), interleukin-17 (IL-17), plasminogen activator inhibitor type 1 (TPAI-1), matrix metallopeptidase-9 (MMP-9), soluble cell adhesion molecules (SVCAM-1, SICAM-1), transforming growth factor alpha (TGF-alpha), monocyte chemotactic protein-1 (MCP-1), interleukin-1alpha (IL-1alpha), interleukin-1beta (IL-1beta), interleukin-9 (IL-9). Levels of every parameter compared between groups. Statistic method of Mann-Whitney test used.

## Results

It was revealed statistically significantly lower level of IFN-g (z=2,60; p,0,001), IL-17 (z=2,78; p<0,001), TPAI-1 (z=2,19; p<0,01), MMP-9 (z=2,60; p<0,02), SICAM-1 (z=2,76; p<0,001) and TGF-alpha (z=2,52; p<0,001) in migraine group. Evaluation of Area Under Curve (AUC) using Receiver Operating Characteristic (ROC) method showed no significant difference in migraine and control groups (AUC=0,63-0,68 in mentioned above parameters) .

## Conclusion

It was statistically significant difference of cytokine levels in migraine patients and control group. However, it does not allow interpreting findings in terms of clinical screening

No conflict of interest.

